# Exploring the relationship between abnormally high expression of *NUP205* and the clinicopathological characteristics, immune microenvironment, and prognostic value of lower-grade glioma

**DOI:** 10.3389/fonc.2023.1007198

**Published:** 2023-05-22

**Authors:** Wenjia Liang, Chenchen Hu, Qingyun Zhu, Xingbo Cheng, Shanjun Gao, Zhendong Liu, Hongbo Wang, Pengxu Li, Yanzheng Gao, Rongjun Qian

**Affiliations:** ^1^ People’s Hospital of Henan University, Henan Provincial People’s Hospital, Microbiome Laboratory, Zhengzhou, Henan, China; ^2^ Intensive Care Unit, Hubei Cancer Hospital, Tongji Medical College, Huazhong University of Science and Technology, Wuhan, Hubei, China; ^3^ Department of Surgery of Spine and Spinal Cord, Henan Provincial People’s Hospital, People’s Hospital of Zhengzhou University, People’s Hospital of Henan University, Zhengzhou, Henan, China; ^4^ Microbiome Laboratory, Henan Provincial People’s Hospital, Henan University People’s Hospital, Zhengzhou, China; ^5^ Department of Urology, Lanzhou University Second Hospital, Lanzhou, Gansu, China; ^6^ Department of Neurosurgery, Henan Provincial People’s Hospital, People’s Hospital of Henan University, People’s Hospital of Zhengzhou University, Zhengzhou, Henan, China

**Keywords:** NUP205, lower-grade glioma, prognosis, pathogenic gene, immunotherapeutic target

## Abstract

Nuclear pore complex (NPC) is a major transport pivot for nucleocytoplasmic molecule exchange. Nucleoporin 205 (NUP205)—a main component of NPC—plays a key regulatory role in tumor cell proliferation; however, few reports document its effect on the pathological progression of lower-grade glioma (LGG). Therefore, we conducted an integrated analysis using 906 samples from multiple public databases to explore the effects of *NUP205* on the prognosis, clinicopathological characteristics, regulatory mechanism, and tumor immune microenvironment (TIME) formation in LGG. First, multiple methods consistently showed that the mRNA and protein expression levels of *NUP205* were higher in LGG tumor tissue than in normal brain tissue. This increased expression was mainly noted in the higher WHO Grade, IDH-wild type, and 1p19q non-codeleted type. Second, various survival analysis methods showed that the highly expressed *NUP205* was an independent risk indicator that led to reduced survival time of patients with LGG. Third, GSEA analysis showed that *NUP205* regulated the pathological progress of LGG *via* the cell cycle, notch signaling pathway, and aminoacyl-tRNA biosynthesis. Ultimately, immune correlation analysis suggested that high *NUP205* expression was positively correlated with the infiltration of multiple immune cells, particularly M2 macrophages, and was positively correlated with eight immune checkpoints, particularly PD-L1. Collectively, this study documented the pathogenicity of *NUP205* in LGG for the first time, expanding our understanding of its molecular function. Furthermore, this study highlighted the potential value of *NUP205* as a target of anti-LGG immunotherapy.

## 1 Introduction

Glioma, which originates from neural stem cells or progenitor cells, is the most common primary malignancy of the intracranial brain parenchyma (1). According to the World Health Organization (WHO) 2016 classification system, gliomas are generally divided into two types: WHO Grade I to III, considered as lower-grade gliomas (LGGs), and WHO Grade IV, considered as glioblastomas (GBMs) (2). LGG accounts for approximately 43.2% of all gliomas and has a median survival rate of 7 years (3, 4). However, due to the invasive growth characteristic of LGG cells, LGGs exhibit a high recurrence rate, and 70% of LGGs eventually progress to high-grade malignancies within 5–10 years (5). The current mainstay treatment for patients with LGG is surgical resection and adjunct radiotherapy combined with chemotherapy; however, their therapeutic effects do not meet ideal expectations (6). Fortunately, cancer research has expanded from solely studying the molecular event changes of tumor cells to studying the overall abnormality of the tumor immune microenvironment (TIME), allowing the advent of new immunotherapies that provide precise, individualized cancer treatment (7). However, current immunotherapeutic strategies are limited to only a subset of cancer patients, as only 20% of patients respond to therapy (8). Thus, finding more targets to mediate the immune response in the LGG TIME is crucial for prolonging the life of patients with LGG.

Abnormal export of pathogenic mRNAs from the nucleus increases the malignant biological behavior of cancer cells, which accelerates their malignant progression (9). The nuclear pore complex (NPC) is the major transport pivot for nucleocytoplasmic molecule exchange, including mRNAs. By controlling mRNA export from the nucleus, the NPC regulates vital activities of cells, such as cell growth, differentiation, apoptosis, and immune response (9–11). Recently, an increasing body of evidence showed that the NPC is closely related to tumorigenesis and regulates immune response in the tumor microenvironment (TME). For example, overexpression of *NUP37* activates the PI3K/AKT/mTOR signaling pathway promoting proliferation, migration, and invasion of gastric cancer cells, and also gives rise to an immunosuppressive microenvironment in gliomas (12, 13). *NUP205*, as a core subunit of the NPC, is responsible for the gate control function and long-term maintenance of NPC, which is of great significance to the normal progress of cell life activities (14, 15). Thus, many scientists have studied the role of abnormal *NUP205* in cancers and found that *NUP205* is also closely related to tumorigenesis. For example, the high expression of *NUP205* increases the proliferation ability of hepatocellular carcinoma cells, leading to poor prognosis in these patients (16, 17). The expression of *NUP205* in nasopharyngeal carcinoma tumor tissue was significantly upregulated compared with that in adjacent tissue, which was an important factor leading to the proliferation of nasopharyngeal carcinoma cells (18). *NUP205* has also been reported to be an oncogene in colorectal cancer, bladder cancer, and lung cancer (19–21). However, the role of *NUP205* in the pathological process of LGG remains elusive, especially in the TIME of LGG. Therefore, this study, for the first time, revealed the relationship between *NUP205* and the prognosis of LGG patients, as well as the TIME of LGG.

This study attempts to reveal the function of *NUP205* in the malignant pathological progression of LGG through mutual validation from multiple databases. Our study is the first to document high *NUP205* expression in LGG using public databases, and the results were confirmed by our basic experiments. We found that aberrantly expressed *NUP205* had a profound impact on the prognosis of patients with LGG by data analysis of 903 cases from two datasets, which suggested that *NUP205* could be a potential biomarker for LGG. At the same time, we explored the mechanism of the overexpression of *NUP205* in LGG by documenting the relationship between *NUP205* and its DNA methylation. Ultimately, we found that *NUP205* might play an important role in the immunosuppressive microenvironment of LGG, which could provide a theoretical basis for the use of *NUP205* as an immunotherapeutic target.

## 2 Materials and methods

### 2.1 Data collection and tissue preparation

In this study, Gene Expression Profiling Interactive Analysis (GEPIA, http://gepia.cancer-pku.cn/), an online bioinformatics analysis tool (22), was used to explore the mRNA expression of *NUP205* in LGG. We collected data from the Gene Expression Omnibus (GEO, https://www.ncbi.nlm.nih.gov/geo/) database to verify the results of the GEPIA database (GSE12657: 13 LGG tissues vs 5 control tissues; GSE21354: 10 LGG tissues vs 4 normal brain tissues; GSE70231: 24 LGG tissues vs 6 normal brain tissues) (23–25). In the database of The Cancer Genome Atlas (TCGA, https://portal.gdc.cancer.gov/) (26), we collected RNA sequencing data with the corresponding clinical information of 503 patients with LGG and the DNA methylation sequencing data of 511 patients with LGG, which were used to explore the effects of *NUP205* on the prognosis, clinicopathological characteristics, regulatory mechanism, and TIME in LGG. The detailed patient information of the TCGA RNA-seq database is presented in **Supplementary Table S1**. Finally, we collected the RNA sequencing data of 403 LGG tissue samples in the Chinese Glioma Genome Atlas (CGGA, http://www.cgga.org.cn/) database to verify the results obtained from the TCGA RNA-seq database (27). The detailed patient information of the CGGA RNA-seq database is presented in **Supplementary Table S2**.

Five brain tissue samples from epilepsy patients and five tumor tissue samples from patients with LGG were obtained from the operating room of Henan Provincial People’s Hospital and used to determine the change of *NUP205* mRNA expression in LGG; the histopathology and IDH mutation status of these five glioma patients is shown in **Supplementary Table S3**. Then, three additional brain tissue samples from epilepsy patients and three tumor tissue samples from patients with LGG were used to detect the change in protein expression of NUP205, CD163, and CD274. Finally, we collected three glioma tissues of WHO Grade III and three glioma tissues of WHO Grade II and compared the protein expression of NUP205 between them. All participating patients provided informed consent, and the project was approved by the ethics committee of Henan Provincial People’s Hospital (Ethics approval number: 2020107).

### 2.2 Culture of human astrocyte cells and glioma cells

Human astrocyte (HA) and glioma cell lines (SHG44, T98, and LN229) were purchased from the Cell Bank of the Chinese Academy of Sciences and Qingqi Cell Bank (Shanghai, China), respectively. The cells in both groups were grown in Dulbecco’s Modified Eagle Medium culture (Procell, China) with 10% fetal bovine serum (Gibco, USA) and 1% penicillin (Procell, China) and incubated at 37°C and 5% carbon dioxide. After cells were grown to confluence in 60-mm dishes, total RNA was extracted by RT-qPCR to compare the expression levels of *NUP205* in normal astrocytes and glioma cells. To demonstrate whether *NUP205* mRNA expression in glioma cells is regulated by DNA methylation, we added 100 µM ademetionine disulfate tosylate (SAM) (Topscience, China) and treated them with SHG44, T98, and LN229 for 10 h. After culturing with SAM, glioma cells were used to identify changes in *NUP205* expression after DNA hypermethylation.

### 2.3 Extraction of total RNA and RT-qPCR

According to the protocol of Total RNA Kit I (Omega, USA), we obtained total RNA from cells and tissue samples, and then determined total RNA concentration using a NanoDrop machine (Thermo, USA). Total RNA was reverse transcribed to synthesize cDNA according to the instructions of the NovoScript Plus All-in-one 1st Strand cDNA Synthesis SuperMix kit (Novoprotein, China). cDNA was then amplified by the StepOne Plus Real-Time PCR System (Thermo, USA) according to the instructions of NovoStart^®^ SYBR qPCR SuperMix Plus (Novoprotein, China). The internal-reference gene, *18S*, was selected to standardize the *NUP205* expression. The specific primer sequences of *18S* and *NUP205* were as follows: *18S* forward: 5’-GTAACCCGTTGAACCCCATT-3’ and *18S* reverse: 5’-CCATCCAATCGGTAGTAGCG-3’. *NUP205* forward: 5’-CATCACCCAGAAGGAGCAAG-3’ and *NUP205* reverse: 5’-GGAGTCCCAGAATCACCACA-3’.

### 2.4 Immunohistochemistry

Immunohistochemistry (IHC) staining was performed following a standard procedure. After brain tissue samples were sliced into 5.0-µm paraffin sections, the sections were deparaffinized in xylene and dehydrated in graded concentrations of ethanol. After EDTA (ZSGB-BIO, China) antigen retrieval and quenching of endogenous peroxidase, 10% bovine serum albumin solution was used to block non-specific antigens for 1 h at 30 °C. These sections were incubated with 1:200 Anti-NUP205 (Proteintech, China), 1:250 Anti-CD163 (Invitrogen, USA), or 1:250 Anti-CD274 (Proteintech, China) overnight at 4 °C, respectively. Subsequently, the incubation of secondary antibodies and DAB color development were completed following the instructions of the Mouse/Rabbit enhanced polymer detection system (ZSGB-BIO, China). Finally, the stained area was photographed under a 200× microscope, and the results were calculated by ImagePro-Plus software (version 6.0).

### 2.5 Western blot

An appropriate amount of brain tissue was homogenized with RIPA lysate (EpiZyme, China) and a protease inhibitor (EpiZyme, China). The brain tissue was then split on ice for 30 min, and the protein supernatant was centrifuged at 12000 rpm for 30 min at 4 °C. The protein concentration was detected using a BCA kit (Biosharp, China). Briefly, the protein was boiled at 100 °C for 10 min in 4× loading buffer (Solarbio, China), separated by SDS-PAGE electrophoresis, and then transferred to a PVDF membrane (Bio-Rad, USA). After the membrane was sealed with 5% evaporated milk, NUP205 (1:1000; Proteintech, China) and β-actin (1:1000; Bioss, China) primary antibodies were added overnight at 4 °C. Then, the membrane was incubated in goat anti-rabbit IgG H&L antibody (1:2000; Bioss, China) at 37 °C for 1 h. Finally, the protein blots were developed with a chemiluminescence reagent kit (Beyotime Biotechnology), and ImagePro-Plus software (version 6.0) was used for the quantitative analysis.

### 2.6 Meta-analysis of *NUP205* in LGG

In this study, a meta-analysis was used to explore the effect of *NUP205* on the OS of patients with LGG. First, after searching PubMed databases, we found a few studies on the relationship between *NUP205* and the OS of patients with LGG. Therefore, we had to assemble 924 samples from three datasets (TCGA RNA-seq database: 503 LGG samples, CGGA RNA-seq database: 403 LGG samples, and GSE43378: 18 LGG samples) to perform a meta-analysis. Subsequently, we adopted the Cox analysis model on the three datasets separately to calculate the hazard ratio (HR) of *NUP205* on the prognosis of patients with LGG using R software (version 4.0.3). Meanwhile, the Q test (I^2^ statistics) was performed to evaluate the heterogeneity of the three datasets. Ultimately, we selected a random effect model according to I^2^ > 50% and *p* < 0.05 to calculate combined HR and a 95% confidence interval (95% CI).

### 2.7 Correlation analysis between *NUP205* and TIME in LGG

Tumor Immune Estimation Resource (TIMER, https://cistrome.shinyapps.io/timer/) is a common platform for analyzing the relationship between immune cell infiltrates and multiple variables based on the TCGA samples (28). Therefore, we utilized the TIMER database to analyze the relationship between *NUP205* and TIME in LGG. First, we explored the correlation between the expression of *NUP205* and six immune infiltrates (B cells, CD4+ T cells, CD8+ T cells, neutrophils, macrophages, and dendritic cells). Second, Kaplan–Meier analysis was used to reveal the effects of *NUP205* expression and the six immune infiltrates on the OS of patients with LGG. Finally, we analyzed the correlation between the types of somatic copy number alteration (SCNA) of *NUP205* and the six immune infiltrates.

The TIMER database cannot analyze the relationship between target genes and immune cell subtypes. For this reason, we used data from the TCGA RNA-seq database to further understand the relationship between the expression of *NUP205* and various immune cell subtypes. The “CIBERSORT” package on R software (version 4.0.3) was performed to calculate and visualize the effects of *NUP205* expression on infiltrates of various immune-cell subtypes. Spearman analysis was used to reveal the correlation between *NUP205* expression and markers of various immune-cell subtypes. Immune checkpoint therapy has now become a hotspot for antitumor therapy, and thus, we used Pearson analysis on R software (version 4.0.3) to calculate the correlation between *NUP205* expression and the 8 famous immune checkpoints (*CD274*, *PDCD1*, *HAVCR2*, *CD96*, *KLRB1*, *IDO1*, *CD276*, *LAG3*) based on the TCGA database.

### 2.8 Gene set enrichment analysis of the high *NUP205* phenotype in LGG

Gene set enrichment analysis (GSEA) is a common tool used to arrange the position of target genes into a preconstructed functional gene set to predict their function (29). Therefore, we used GSEA software (version 4.0) to predict the cell signaling pathways through which *NUP205* regulates LGG. First, according to the median value of *NUP205* expression level in LGG, we divided the data from the TCGA RNA-Seq database into high *NUP205* phenotype and low *NUP205* phenotype. Then, we permuted the high *NUP205* phenotype 1000 times, and hundreds of cell signaling pathways emerged. Finally, according to nominal (NOM) p < 0.05 and false discovery rate (FDR) Q < 0.25, we selected the cell signaling pathways where a high *NUP205* phenotype might influence LGG.

### 2.9 Statistical analysis

The results of RT-qPCR, IHC staining, and western blotting were calculated by unpaired t-tests on GraphPad Prism 9 (San Diego, CA, USA) software. The chi-squared test was used to compare *NUP205* expression between normal brain and LGG tissue, as well as to analyze the relationship between the expression of *NUP205* and several clinical features. Kaplan–Meier analysis was used to document the correlation between *NUP205* expression and the OS of patients with LGG. Receiver operating characteristic (ROC) curves were used to calculate the diagnostic value of *NUP205* in patients with LGG. Cox regression analysis was used to determine whether the high expression of *NUP205* was a risk factor for patients with LGG. Pearson analysis was used to search for the co-expressed genes of *NUP205*. All results were derived by R software (version 4.0.3), and *p* < 0.05 was considered statistically significant.

## 3 Results

### 3.1 Significantly increased *NUP205* in the transcriptome and proteome of LGG

**Figure 1:**
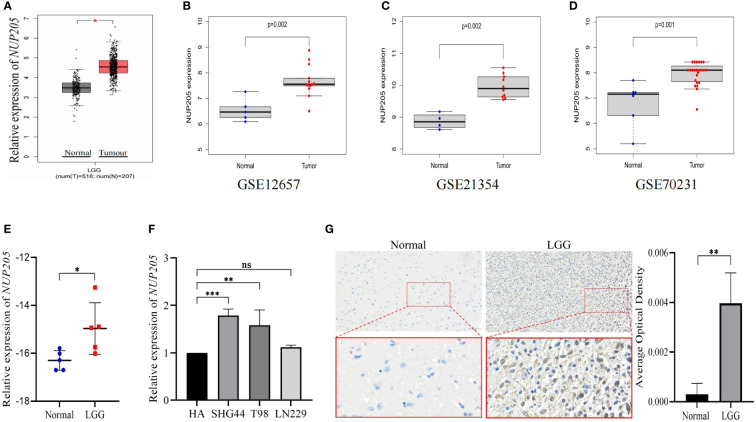
The mRNA and protein expression of *NUP205* was increased in LGG. The results of **(A)** GEPIA, **(B)** GSE12657, **(C)** GSE21354, and **(D)** GSE70231 database showed that the mRNA expression of *NUP205* increased in LGG tumor tissues. **(E)** The results of RT-qPCR showed that the mRNA expression of *NUP205* was increased in LGG tissue. **(F)** The results of RT-qPCR showed that the mRNA expression of *NUP205* was increased in glioma cells. **(G)** The results of IHC staining showed that the protein expression of NUP205 was increased in LGG tissue. ns, no statistically significant; *: *p*<0.05, **: *p*<0.01, ***: *p*<0.001. *p*<0.05 was considered statistically significant.

The association between the clinical progression of cancer and the high expression of pathogenic genes prompted us to report the expression of *NUP205* in patients with LGG. First, the results of both the GEPIA database and chi-squared test based on the GEO databases (GSE12657, GSE21354, GSE70231) showed that *NUP205* mRNA expression was higher in LGG than in normal brain tissue (**Figures 1A–D** respectively). These results were subsequently verified with RT-qPCR analysis (**Figures 1E, F**). Since the protein serves as the last unit to perform certain biological functions, we performed IHC staining on NUP205 and showed significantly increased protein expression in the LGG tissue compared to the controls (**Figure 1G**). Taken together, these results show that *NUP205* had abnormally high expression in LGG compared with the normal brain, implying that *NUP205* might play an important role in the pathological progression of LGG.

### 3.2 Relationship between *NUP205* expression and clinical characteristics in LGG

**Figure 2:**
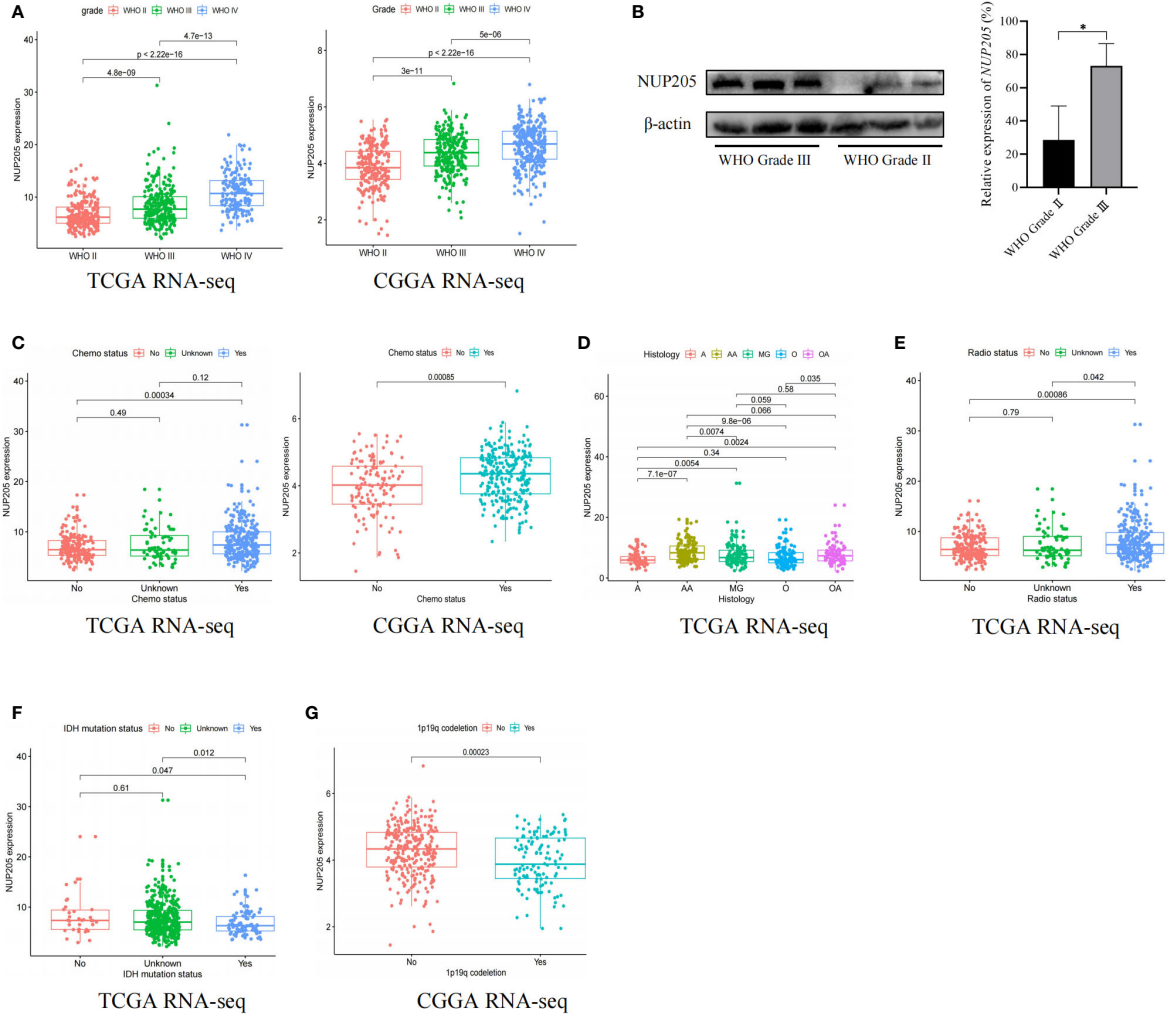
The relationship between the expression of *NUP205* and clinical features in LGG. **(A)** WHO grade, **(B)** Western blot results showed that the protein expression of NUP205 was significantly higher in WHO grade III than in WHO grade II. **(C)** Chemotherapy status, **(D)** Histology, **(E)** Radiotherapy status, **(F)** IDH-mutation status, **(G)** 1p19q codeletion status. *p<0.05. *p*<0.05 was considered statistically significant.

It is well known that high pathogenic gene expression is often concomitant with malignant clinical characteristics in cancer. This highlights the importance of exploring the relationship between *NUP205* expression and the clinical characteristics of patients with LGG. First, we found that the mRNA expression of *NUP205* increased with WHO Grade based on the TCGA RNA-seq and CGGA RNA-seq databases (**Figure 2A**). In addition, the results of western blotting showed that the protein expression of *NUP205* in the WHO Grade III tumor tissue was significantly higher than that of WHO Grade II tissue (**Figure 2B**). Second, based on the TCGA and CGGA databases, we found that the expression of *NUP205* in chemotherapy-type patients was significantly higher than that of non-chemotherapy types (**Figure 2C**). Third, analysis of the histological types of LGG from the TCGA RNA-seq database showed that *NUP205* expression is highest in anaplastic astrocytoma (AA) and lowest in astrocytoma (A) (**Figure 2D**). Fourth, we found that *NUP205* expression was higher in the radiotherapy type and IDH-wild type LGG compared to the non-radiotherapy type and IDH-mutated type based on the TCGA RNA-seq database (**Figures 2E, F**). Finally, we found that the expression of *NUP205* was higher in the 1p19q non-codeleted type than in the 1p19q codeleted type LGG based on the CGGA RNA-seq database (**Figure 2G**). In short, the above results suggested that the high expression of *NUP205* always appeared in the malignant clinical subtypes of LGG, implying its effect on poor prognosis in patients with LGG.

### 3.3 High *NUP205* expression led to poor prognosis in patients with LGG and was an independent risk factor for LGG

**Figure 3:**
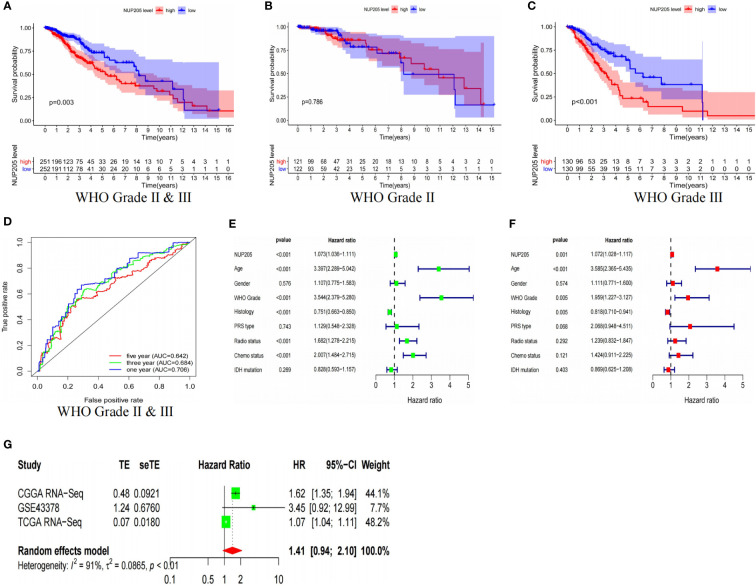
The results based on TCGA RNA-seq database showed that the high expression of *NUP205* leads to poor prognosis of LGG patients. The result of **(A)** Kaplan-Meier analysis for LGG patients of WHO Grade II & III, **(B)** Kaplan-Meier analysis for LGG patients of WHO Grade II, **(C)** Kaplan-Meier analysis for LGG patients of WHO Grade III, **(D)** ROC curve, **(E)** Univariate analysis, **(F)** Multivariate analysis. **(G)** Meta analysis. *p*<0.05 was considered statistically significant.

To find more evidence to support the correlation between *NUP205* and the prognosis of patients, we conducted Kaplan–Meier analysis, ROC curves analysis, univariate and multivariate analysis, and meta-analysis based on the TCGA and CGGA databases. First, Kaplan–Meier analysis showed that LGG patients of WHO Grades II & III with high *NUP205* expression had shorter OS (**Figure 3A** and **Supplemental Figure S1A**). For the LGG patients of WHO Grade II, the Kaplan–Meier analysis had similar results in the CGGA database (**Supplemental Figure S1B**), while there was no statistical significance in the TCGA database (**Figure 3B**). LGG patients of WHO Grade III with high *NUP205* expression were found to have significantly lower OS than those with low expression (**Figure 3C** and **Supplemental Figure S1C**). These results suggest that high *NUP205* expression might lead to a poor prognosis in LGG patients, whether they are of WHO Grade II or III. Subsequently, to explore the diagnosis value of *NUP205* for patients with LGG, we constructed ROC curves and found that *NUP205* can be used as a biomarker for LGG diagnosis (**Figure 3D** and **Supplemental Figure S1D**). In addition, the results of univariate and multivariate analysis showed that high *NUP205* expression was HR > 1 (p < 0.05) in LGG, which suggests its role as an independent risk factor for patients with LGG (**Figures 3E, F**, and **Supplemental Figures S1E, F**). Finally, the results of the meta-analysis showed that the HR and 95% CI were 1.41 and 0.94–2.10, respectively (**Figure 3G**). In summary, all evidence shows that *NUP205* is a pathogenetic gene for LGG and leads to a poor prognosis in patients with LGG.

### 3.4 DNA methylation of *NUP205* was negatively correlated with its mRNA expression and influenced the prognosis of patients with LGG

**Figure 4:**
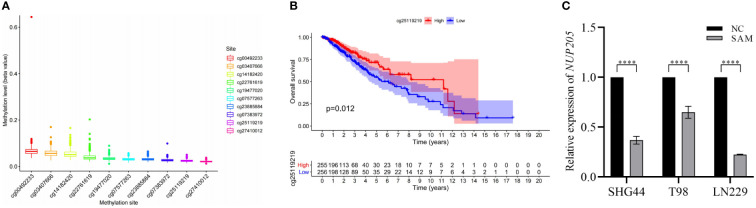
The relationship between the mRNA expression of *NUP205* and its DNA methylation in LGG. **(A)** DNA methylation sites of *NUP205*. **(B)** The result of Kaplan-Meier analysis showed that hypermethylation of cg25119219 led to reduce the overall survival of LGG patients. **(C)** The result of RT-qPCR showed that the expression of *NUP205* was decreased in glioma cells treated with SAM. ns, no statistically significant; ****: *p*<0.0001. *p*<0.05 was considered statistically significant.

Previous studies reported that DNA methylation could negatively regulate the expression of pathogenic genes in LGG (30), therefore, we tried to reveal the mechanism through which DNA methylation regulates *NUP205* expression. First, using the TCGA database to extract DNA methylation data, we screened ten methylation sites that may regulate *NUP205* expression (**Figure 4A**). Subsequently, Kaplan–Meier analysis was used to analyze whether these methylation sites were related to the prognosis of LGG patients. We found that LGG patients with hypermethylation of cg25119219 had longer OS than those with hypomethylation of cg25119219 (**Figure 4B**). Finally, SAM, a drug used to promote DNA methylation, was cultured with glioma cells (SHG44, T98, and LN229). The RT-qPCR results showed that *NUP205* expression significantly decreased in glioma cells cultured with SAM (**Figure 4C**). Therefore, we speculated that the abnormally high expression of *NUP205* might result from its DNA demethylation, and cg25119219 could be used as a biomarker for LGG patients.

### 3.5 Co-expression analysis of *NUP205* and GSEA analysis of *NUP205* in LGG

**Figure 5:**
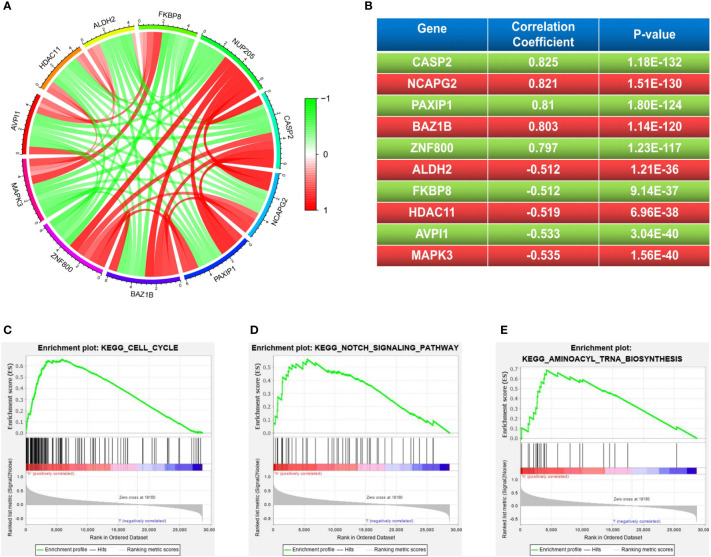
**(A, B)** The 5 most positive and negative genes related to *NUP205* in LGG. The results of GSEA analysis suggested that *NUP205* participated in the pathological process of LGG *via* **(C)** cell cycle, **(D)** notch signaling pathway, **(E)** aminoacyl-tRNA biosynthesis. *p*<0.05 was considered statistically significant.

The co-expression of pathogenic genes may exert similar functions to synergistically regulate cancer progression (31). Therefore, to better understand the role that *NUP205* plays in the pathological progression of LGG, we performed co-expression analyses of *NUP205* based on the TCGA database. We picked out the five most positively correlated genes (*CASP2*, *NCAPG2*, *PAXIP1*, *BAZ1B*, *ZNF800*) and the five most negatively correlated genes (*MAPK3*, *AVPI1*, *HDAC11*, *FKBP8*, *ALDH2*) (**Figures 5A, B**). By reviewing previous studies, we found that *CASP2*, *NCAPG2*, *BAZ1B*, and *ZNF800*, the genes most positively related to *NUP205*, were reported as carcinogenic genes in cancer, especially *CASP2*, *NCAPG2*, and *BAZ1B*, which were found to be carcinogenic genes in glioma (32–35). However, the genes most negatively associated with *NUP205* were reported to be tumor suppressor genes, such as *AVPI1*, *HDAC11*, *FKBP8*, and *ALDH2* (36–39). Therefore, the results of the co-expression analyses also supported our view that *NUP205* is a pathogenic gene of LGG.

To better understand the molecular mechanism of *NUP205* in the pathological progression of LGG, we used GSEA analysis to explore the cell signaling pathways that *NUP205* might act through in LGG. Our results showed that high *NUP205* expression was significantly enriched in the cell cycle, notch signaling pathway, and aminoacyl-tRNA biosynthesis (**Figures 5C–E** and **Supplementary Table S4**). Previous studies have documented the effect of the aforementioned pathways in LGG progression, such as malignant proliferation of tumor cells and immune evasion (40, 41). Therefore, we speculated that the high expression of *NUP205* might promote the malignant biological behavior of LGG and induce the formation of the LGG immunosuppressive microenvironment through these pathways.

### 3.6 Association of *NUP205* with immune cell infiltration and immune checkpoints in LGG

Previous studies have shown that pathogenic genes can shape the immunosuppressive microenvironment of cancer (42). Also, the results of GSEA suggested that *NUP205* might promote the immunosuppressive microenvironment of LGG. Thus, these hints inspired us to explore the role of *NUP205* in the TIME of LGG.

**Figure 6:**
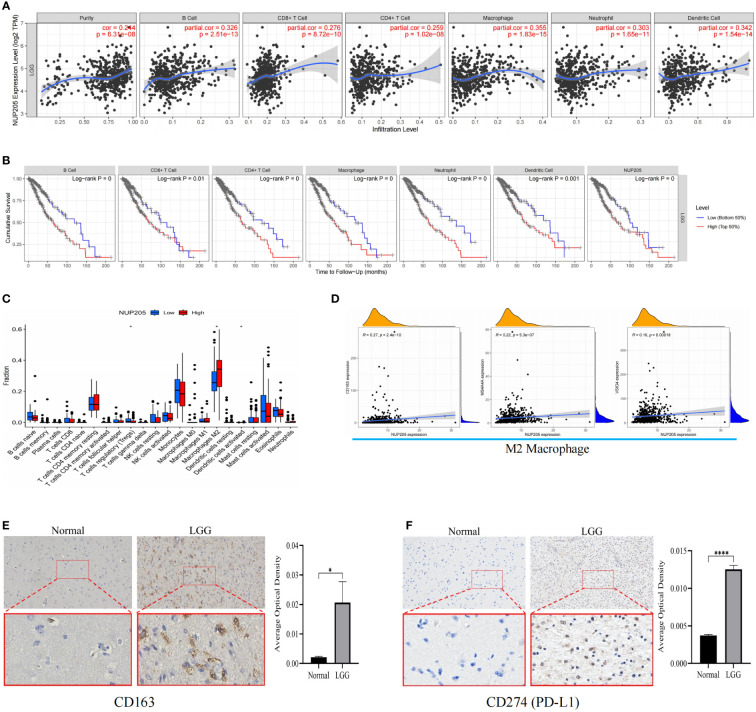
**(A)** The results of TIMER database showed that *NUP205* expression was positively correlated with six immune infiltrations (B cell, CD4 + T cell, CD8 + T cell, neutrophil, macrophage, and dendritic cell). **(B)** The results of Kaplan-Meier analysis showed that the high level of six immune infiltration and high expression of *NUP205* lead to poor prognosis of LGG patients. **(C)** The results of “CIBERSTART” analysis. **(D)** The results of Pearson analysis showed that *NUP205* was positively correlated with the markers of M2 macrophages (*CD163*, *VSIG4*, *MS4A4A*). **(E)** The IHC staining of CD163 showed that the protein expression of CD163 in LGG tumor tissue was higher than that in control brain tissue. **(F)** The IHC staining of PD-L1 showed that the protein expression of PD-L1 in LGG tumor tissue was higher than that in control brain tissue. ns, no statistically significant; *: *p*<0.05, ****: *p*<0.0001. *p*<0.05 was considered statistically significant.

The TIMER database was used to explore the relationship between *NUP205* expression and immune infiltration in LGG, and we found that *NUP205* expression positively correlated with infiltration of six types of immune cells (B cells, CD4^+^ T cells, CD8^+^ T cells, neutrophils, macrophages, and dendritic cells) (**Figure 6A**). Subsequently, the Kaplan–Meier analysis showed that both high infiltration of the six immune cells and high expression of *NUP205* led to a shorter OS in patients with LGG (**Figure 6B**). Finally, we found that arm-level gain and arm-level deletion of *NUP205* in LGG led to higher infiltration levels of 5 immune cells (B cells, CD8^+^ T cells, neutrophils, macrophages, and dendritic cells) (**Figure S2A**). Taken together, the above results suggest that high *NUP205* expression is positively correlated with the infiltration of immune cells, thereby leading to poor prognosis in LGG patients.

Since the TIMER database lacks analysis between the target gene and the immune cell subtypes, we used the “CIBERSTART” analysis to explore the relationship between *NUP205* expression and the infiltration levels of 22 immune cell subtypes based on the TCGA database. We found that the *NUP205* expression in multiple immune cell subtypes was markedly increased in LGG, particularly M2 macrophages (**Figure 6C**). Surprisingly, the correlation analysis also showed that *NUP205* expression was positively correlated with markers of M2 macrophages (*CD163*, *VSIG4*, *MS4A4A*) and negatively correlated with those of M1 macrophages (**Figure 6D** and **Table 1**). Finally, we performed IHC staining for CD163 (a M2 macrophage marker) and found that CD163 expression, like NUP205, was significantly increased in LGG compared to control brain tissue (**Figure 6E**). In summary, these results show that the expression of *NUP205* is positively correlated with M2 macrophages, suggesting that *NUP205* might be related to the infiltration of M2 macrophages in the TIME of LGG.

**Table 1:** Results of correlation analysis between *NUP205* and multiple immune-cell markers based on TCGA database.

Immune Cell	Marker	Correlation Coefficient	*p*-value
B cell	CD19	0.042056185	0.334328843
	CD79A	0.043779243	0.314776989
CD8+ T cell	CD8A	-0.097166164	0.025462501
	CD8B	0.016937164	0.697526815
CD4+ T cell	CD4	0.135468796	0.00180596
M1 macrophage	NOS2	-0.030512764	0.483617805
	PTGS2	-0.030952767	0.477331716
M2 macrophage	CD163	0.271946	2.39E-10
	VSIG4	0.162536924	0.000176996
	MS4A4A	0.216558638	5.27E-07
Neutrophil	ITGAM	0.093711881	0.031192334
	CCR7	0.09180486	0.034774328
Dendritic cell	HLA-DQB1	0.138549547	0.001413408
	HLA-DRA	0.19963044	3.88E-06
	HLA-DPA1	0.181794674	2.68E-05
	NRP1	0.376177016	0

Immune checkpoint treatment has become a hotspot in cancer treatment. Therefore, we explored the relationship between *NUP205* and 8 famous immune checkpoints in LGG by Spearman analysis based on the TCGA database. We found that *NUP205* expression positively correlated with the eight immune checkpoints (*CD274*, *PDCD1*, *HAVCR2*, *CD96*, *KLRB1*, *IDO1*, *CD276*, *LAG3*) (**Table 2**). Surprisingly, CD274 (PD-L1), the most famous immune checkpoint marker, expression was significantly increased in LGG, similar to that of *NUP205* (**Figure 6F**). In conclusion, *NUP205* might be a potential target for LGG immunotherapy because it is positively correlated with the expression of various immune checkpoints, especially PD-L1.

**Table 2:** Results of correlation between *NUP205* and multiple immune-checkpoints based on TCGA database.

Checkpoint	Correlation Coefficient	*p*-value
CD274	0.11509807	0.00808293
PDCD1	0.20471315	2.06E-06
HAVCR2	0.15116834	0.000492
CD96	0.17705533	4.22E-05
KLRB1	0.17801064	3.83E-05
IDO1	0.24566005	1.04E-08
CD276	0.45277369	0
LAG3	0.16058236	0.00021204

## 4 Discussion

NPC can not only regulate transport of nucleocytoplasmic molecules, but can also control DNA damage repair and chromatin translocation and silencing, making it relevant to the pathological progression of cancers (43). Since *NUP205* preserves the integrity of NPC, recent studies have focused on revealing its role in various malignancies. For example, LncRNA HOTAIR can upregulate the expression of *NUP205* to increase the growth, migration, and invasion of papillary thyroid carcinoma cells *via* absorbing the role of miR-488-5p (44). The overexpression of *NUP205* can accelerate the cell cycle, increasing the proliferation of acute myoid leukemia cells (45). Unfortunately, few reports on the role of *NUP205* in the malignant process of LGG exist. Existing studies have all reported that *NUP205* is an oncogene in several cancers, leading us to believe that *NUP205* may also be a pathogenic gene in LGG.

In this study, we attempted to reveal the influence of *NUP205* expression on the pathological progression of LGG. First, we found that the mRNA expression of *NUP205* was significantly increased in LGG tissue using the GEPIA and GEO databases. Our own experimental data verified that both mRNA and protein expression of *NUP205* are increased. These different perspective analyses made our findings more credible. Previous studies have shown that the oncogene is present in a high expression state and promotes the pathological progression of LGG. For example, the oncogene *METTL21B* is highly expressed in LGG tissue and leads to a poor prognosis in patients (46). These findings are highly consistent with our own findings, thereby further proving that *NUP205* is a pathogenic gene in LGG.

To solidify our hypothesis that *NUP205* is in fact a pathogenic gene in the pathological process of LGG, we performed a correlation analysis between *NUP205* and the clinical characteristics of patients with LGG. First, we found that the mRNA and protein expression of *NUP205* in WHO Grade III gliomas was significantly higher than that in WHO Grade II gliomas, and that the prognosis of glioma patients worsens with the improvement of WHO Grade (47). Second, we found that *NUP205* expression was higher in chemotherapy type, IDH wild type, and 1p19q no-codeleted type of LGG, and these clinical features are associated with a worse prognosis (48). After the above discussion, we speculated that highly expressed *NUP205* was closely related to the poor prognosis of patients with LGG. To further support our hypothesis, the Kaplan–Meier analysis showed that patients with LGG with high *NUP205* expression had a poor prognosis, and its increased expression can promote malignant progression of LGG. Previous studies reported similar findings. For example, high *NUP205* expression leads to poor prognosis in patients with hepatocellular carcinoma (17). In addition, Cox regression and meta-analysis further confirmed that *NUP205* is an independent risk factor for LGG, and an indicator of poor prognosis in patients with LGG, respectively. Together, these results demonstrate that *NUP205* is indeed a pathogenic gene for LGG and, as such, may serve as a biomarker for predicting the prognosis of patients with LGG and a potential therapeutic target for the treatment of LGG.

Next, we aimed to explore the reason behind the increased expression of *NUP205* in LGG. The changes in aberrant DNA methylation status often occur during the pathological progression of LGG, and as such, DNA hypomethylation of the target gene can significantly increase its expression level (49). Therefore, we sought to reveal the reason behind the high *NUP205* expression in LGG from the perspective of DNA methylation. We found that *NUP205* expression decreased in glioma cells after treatment with promethylating drugs compared to untreated LGG cells. The expression of *NUP205* in these glioma cells was higher than that in normal human astrocytes. This illustrated that *NUP205* expression was negatively regulated by DNA methylation. Previous studies have reported that the methylation sites of pathogenic genes, such as DNA hypomethylation of *EMILIN2* (50), could serve as markers for predicting the prognosis of patients with LGG. Thus, in this study, we screened ten methylation sites that could affect *NUP205* expression, and by using the Kaplan–Meier analysis method, and we found that the hypomethylation of cg125119219 was closely related to poor prognosis in LGG patients. In short, we found that *NUP205* expression might be negatively regulated by its DNA methylation level in LGG, and that cg125119219 could be used as a biomarker for predicting prognosis in patients with LGG.

Co-expression analysis can be applied to determine the function of target genes by looking up their co-expressed genes (51). Our results suggest that many genes that positively correlate with *NUP205* play a role in the carcinogenesis of glioma. For instance, overexpression of *NCAPG2* can regulate the activation of Wnt/β-catenin pathway to promote proliferation, migration, and invasion of GBM cells, and knockdown of *NCAPG2* inhibited tumorigenesis of GBM *in vivo* (33). Also, the high expression of *BAZ1B* can also increase proliferation, migration, invasion, and inhibition of apoptosis in GBM cells (34). In our study, the *NCAPG2* and *BAZ1B* genes were positively related to *NUP205* and have been reported as oncogenes for glioma. In addition, *HDAC11*, which was negatively correlated with the expression of *NUP205*, was found to have low expression in glioma tissue and was associated with a better prognosis in glioma patients (37). Interestingly, *AVPI1*, *FKBP8*, and *ALDH2*, which were most negatively correlated with *NUP205* in LGG, were all reported to be tumor suppressor genes in cancer (36, 38, 39). This discovery further strengthens our previous view that *NUP205* is a pathogenic gene in LGG.

Exploring the molecular mechanism of *NUP205* in LGG will improve our understanding of the malignant progression of the disease. As such, we performed a GSEA analysis and found that *NUP205* influenced LGG progression *via* the cell cycle, notch signaling pathway, and aminoacyl-tRNA biosynthesis. Many studies have reported that activation of these cell signaling pathways is associated with the malignant progression of cancers. For example, knockdown of *MRPL42* increased the G1 and G2/M phases and decreased the S phase in the cell cycle of glioma cells, suggesting its role in accelerating the malignant advance of gliomas (52). In addition, aminoacyl-tRNA biosynthesis is upregulated in gastric cancer, leading to a poor prognosis seen in patients (53). Interestingly, the activation of the notch signaling pathway is not only able to maintain glioma cells in an aggressive and proliferative state, but is also closely associated with the formation of the TIME of multiple cancers (54, 55). Briefly, *NUP205* may play an important role in LGG malignant progression, including the growth, invasion, and formation of the immunosuppressive microenvironment.

Crosstalk between glioma and immune cells in the TME drives immune cells to reprogram, thereby promoting the transition of TIME to an immunosuppressive microenvironment. Ultimately, cancer cells escape immune cell surveillance, evading immune cell-mediated destruction, and thereby leading to poor prognosis in glioma patients (56, 57). Previous studies have reported that pathogenic genes can participate in regulating the above process (58). Our study showed that *NUP205* acts as a pathogenic gene and may be involved in regulating the immunosuppressive microenvironment of LGG. To prove this, we found that *NUP205* expression positively correlated with the level of six immune-cell infiltrations in LGG TIME. Previous studies have also shown that higher levels of immune cell infiltration in cancer led to worse prognosis in patients (59), which strongly supports our finding that the high level of six immune cell infiltrations led to a short overall survival of patients with LGG. Thus, we speculated that high *NUP205* expression may be involved in the formation of the LGG TIME, which may be one of the important reasons leading to poor prognosis in LGG patients. More importantly, we also found that the expression of *NUP205* was positively correlated with the infiltration of M2 macrophages and negatively correlated with the infiltration of M1 macrophages. Two types of polarization of macrophages exist: classically activated macrophages (M1 macrophages) and alternatively activated macrophages (M2 macrophages) (60). Previous studies reported that tumor-associated macrophages (TAMs) are the most commonly infiltrating immune cells in glioma, and that polarization of TAMs to M2 macrophages led to a poorer prognosis in glioma patients (61). Thus, this finding further strengthened our view that *NUP205* may play an important role in the formation of the TIME in LGG.

The emergence of immunotherapy has provided a new tool for cancer treatment, causing more scientists and researchers to focus on immune-targeted therapy (62). For example, therapeutic anti-CTLA-4 monoclonal antibody drugs have shown remarkable clinical efficacy in treating melanoma (63). In addition, anti-PD-L1 antibody therapy showed good clinical efficacy and superior tolerance in urothelial carcinoma and non-small cell lung cancer (64). Therefore, in the present study, we sought to explore the expression relationship between *NUP205* and multiple immune checkpoint genes and found a positive correlation between *NUP205* and 8 well-known immune-checkpoint genes (*CD274*, *PDCD1*, *HAVCR2*, *CD96*, *KLRB1*, *IDO1*, *CD276*, *LAG3*). We also found that protein expression of NUP205 and PD-L1 (CD274) was indeed positively correlated by IHC staining. Based on the findings that *NUP205* was positively correlated with multiple immune checkpoints in LGG, and that *NUP205* might participate in the formation of the TIME in LGG, we can infer that *NUP205* is a potential immunotherapy target for LGG.

## 5 Conclusions

This study was the first to identify highly expressed *NUP205* as a pathogenic gene and to document its role in the malignant progression and poor prognosis of LGG. More importantly, we found that highly expressed *NUP205* might participate in the formation of the TIME in LGG, leading to a poor prognosis in LGG patients. In summary, this study not only broadened our general understanding of the molecular function of *NUP205* but also proved it as an immunotherapeutic target that may improve the prognosis of patients with LGG.

## Data availability statement

The original contributions presented in the study are included in the article/**Supplementary Material**, further inquiries can be directed to the corresponding author/s.

## Ethics statement

The studies involving human participants were reviewed and approved by the ethics committee of Henan Provincial People’s Hospital (Ethics approval number: 2020107). The patients/participants provided their written informed consent to participate in this study.

## Author contributions

WL, CH, and QZ contributed equally to this work. YG and RQ designed the research. WL and CH revised and wrote the manuscript. QZ and XC performed the experiment. ZL, HW, and PL collected clinical samples. All authors contributed to the article and approved the submitted version.

## Supplementary material

Supplementary Figure 1 The results based on CGGA RNA-seq database showed that the high expression of *NUP205* leads to poor prognosis of LGG patients. The results of **(A)** Kaplan-Meier analysis for LGG patients of WHO Grade II & III, **(B)** Kaplan-Meier analysis for LGG patients of WHO Grade II, **(C)** Kaplan-Meier analysis for LGG patients of WHO Grade III, **(D)** ROC curve, **(E)** Univariate analysis, **(F)** Multivariate analysis. *p*<0.05 was considered statistically significant.

Supplementary Figure 2
**(A)** In LGG, arm-level gain and arm-level deletion of NUP205 led to higher infiltration levels of 5 immune cells **(B)** cell, CD8 + T cell, neutrophil, macrophage, and dendritic cell).
