# Supplementary figures and images for: Exploring the relationship between abnormally high expression of NUP205 and the clinicopathological characteristics, immune microenvironment, and prognostic value of lower-grade glioma
Source: Front Oncol. 2023 May 22;13:1007198. doi: 10.3389/fonc.2023.1007198 (PMC10240054; doi:10.3389/fonc.2023.1007198)

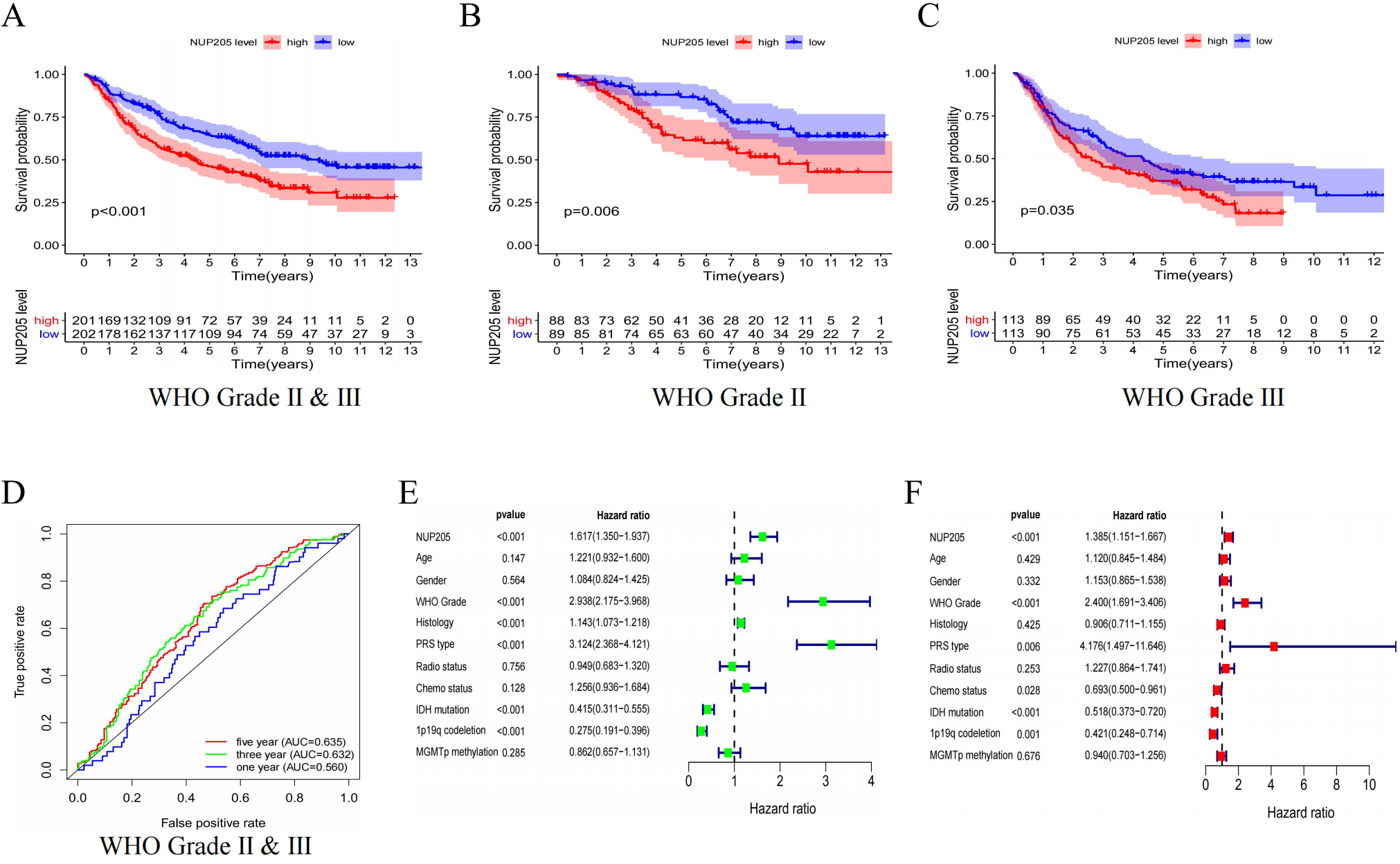

Supplement: Supplementary Figure 1 — The results based on CGGA RNA-seq database showed that the high expression of NUP205 leads to poor prognosis of LGG patients. The results of (A) Kaplan-Meier analysis for LGG patients of WHO Grade II & III, (B) Kaplan-Meier analysis for LGG patients of WHO Grade II, (C) Kaplan-Meier analysis for LGG patients of WHO Grade III, (D) ROC curve, (E) Univariate analysis, (F) Multivariate analysis. p<0.05 was considered statistically significant. [file Image_1.tif]

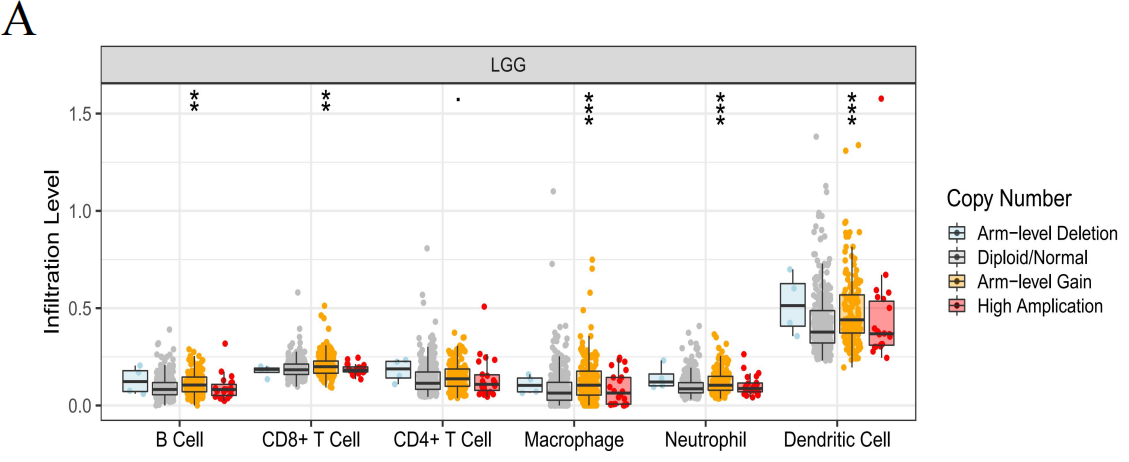

Supplement: Supplementary Figure 2 — (A) In LGG, arm-level gain and arm-level deletion of NUP205 led to higher infiltration levels of 5 immune cells (B) cell, CD8 + T cell, neutrophil, macrophage, and dendritic cell). [file Image_2.tif]
