# Supplementary material for: Exploring the relationship between abnormally high expression of NUP205 and the clinicopathological characteristics, immune microenvironment, and prognostic value of lower-grade glioma
Source: Front Oncol. 2023 May 22;13:1007198. doi: 10.3389/fonc.2023.1007198 (PMC10240054; doi:10.3389/fonc.2023.1007198)
Supplement: Supplementary file 3 [file Table_1.docx]

**Table S1.** **The detailed clinical information of LGG patients in TCGA RNA-seq database**

| **Covariates** | **Type** | **Total** | **Percentages (%)** |
| --- | --- | --- | --- |
| Age | <=41 | 260 | 51.69% |
|  | >41 | 243 | 48.31% |
| Gender | Female | 225 | 44.73% |
|  | Male | 278 | 55.27% |
| WHO Grade | II | 243 | 48.31% |
|  | III | 260 | 51.69% |
| Radio status | No | 187 | 37.18% |
|  | Unknown | 72 | 14.31% |
|  | Yes | 244 | 48.51% |
| Chemo status | No | 167 | 33.20% |
|  | Unknown | 66 | 13.12% |
|  | Yes | 270 | 53.68% |
| PRS type | Primary | 489 | 97.22% |
|  | Recurrent | 14 | 2.78% |
| IDH mutation status | No | 34 | 6.76% |
|  | Unknown | 378 | 75.15% |
|  | Yes | 91 | 18.09% |
| expression | High | 251 | 49.90% |
|  | Low | 252 | 50.10% |
| methylation | High | 251 | 49.90% |
|  | Low | 252 | 50.10% |

**Table S2. The detailed clinical information of LGG patients in CGGA RNA-seq database**

| **Covariates** | **Type** | **Total** | **Percentages (%)** |
| --- | --- | --- | --- |
| PRS type | Primary | 273 | 67.74% |
|  | Recurrent | 130 | 32.26% |
| WHO Grade | II | 177 | 43.92% |
|  | III | 226 | 56.08% |
| Gender | Female | 171 | 42.43% |
|  | Male | 232 | 57.57% |
| Age | <=41 | 222 | 55.09% |
|  | >41 | 181 | 44.91% |
| Radio status | No | 88 | 21.84% |
|  | Yes | 315 | 78.16% |
| Chemo status | No | 134 | 33.25% |
|  | Yes | 269 | 66.75% |
| IDH mutation | No | 100 | 24.81% |
|  | Yes | 303 | 75.19% |
| 1p19q codeletion | No | 280 | 69.48% |
|  | Yes | 123 | 30.52% |
| MGMTp methylation | No | 165 | 40.94% |
|  | Yes | 238 | 59.06% |

**Table S3.** **The detailed clinical information of five tumor tissue samples from patients with LGG for RT-qPCR**

| Patient ID | Gender | Age | WHO Grade | IDH status |
| --- | --- | --- | --- | --- |
| 0002141293 | Male | 44 years old | WHO Grade Ⅲ | mutation |
| 0001873207 | Female | 50 years old | WHO Grade Ⅱ | wild |
| 0002190246 | Female | 36 years old | WHO Grade Ⅱ | wild |
| 0002216937 | Male | 37 years old | WHO Grade Ⅱ | mutation |
| 0002280367 | Male | 55 years old | WHO Grade Ⅲ | wild |

**Table S4. The gene set enriches the high *NUP205* expression based on TCGA RNA-seq database.**

| **Gene set name** | **NES** | **NOM *p*-value** | **FDR q-value** |
| --- | --- | --- | --- |
| Cell cycle | 2.0807445 | 0.003976143 | 0.018471085 |
| Notch signaling pathway | 1.7845356 | 0.0019607844 | 0.05866895 |
| Aminoacyl-tRNA biosynthesis | 1.8640357 | 0.0020449897 | 0.03973896 |

NES: normalized enrichment score; NOM: nominal. Gene sets with NOM *p*-value <0.05 and FDR q-value <0.25 were considered as significantly enriched.
